# Actinomycosis With Pulmonary and Hepatic Involvement: A Case Report and Clinical Insights

**DOI:** 10.7759/cureus.78206

**Published:** 2025-01-29

**Authors:** Rita Vilar da Mota, Ana Rita Oliveira, Patrícia Sobrosa, Sabina Azevedo, Ana R Cambão

**Affiliations:** 1 Internal Medicine, Unidade Local de Saude do Alto Minho, Viana do Castelo, PRT

**Keywords:** actinomyces odontolyticus, actinomycosis, infectious disease, liver actinomycosis, pulmonary actinomycosis

## Abstract

Actinomycosis is a rare, chronic infectious disease caused by Actinomyces spp., characterized by an indolent and slowly progressive course. It represents a diagnostic challenge since its nonspecific clinical features often lead to misdiagnosis, mimicking pathologies such as solid neoplasms, active tuberculosis, nocardiosis, fungal infections, or other granulomatous diseases.

This study describes a 56-year-old male with abdominal and thoracic pain, weight loss, fever, and dyspnea, over a two-week period. Imaging revealed the presence of hepatic abscesses and right-sided pleural effusion. *Actinomyces odontolyticus* was isolated in one sample of blood culture. Management included drainage of the mentioned hepatic and pleural collections, combined with prolonged antibiotic therapy, leading to significant clinical, laboratory, and radiological improvement. This case sheds light on the complexities inherent in diagnosing and treating actinomycosis, underscoring the importance of a multidisciplinary approach in managing complex presentations of this rare disease.

## Introduction

Actinomyces species, named from the Greek words aktinos (meaning 'ray') and mykes (meaning 'fungus') due to their characteristic radial filament arrangement, are anaerobic, Gram-positive, rod-shaped bacteria [1,2].

These microorganisms are predominantly found within the human oropharynx, exhibiting a high concentration in gingival crevices, periodontal pockets, tonsillar crypts, and dental surfaces, including carious lesions and dental plaques [2-7]. Since Actinomyces spp. are widely found in the normal human flora, especially within the oral microbiome, these infections are mainly endogenous [3,4].

Actinomycosis is an uncommon bacterial infection caused by Actinomyces spp., often characterized by indolent progression, nonspecific symptoms, and the propensity to mimic malignancies or other infections, making its diagnosis deeply challenging [8]. The cervicofacial region is the most common site of infection in clinical practice; however, it may also occur in the abdominal, thoracic, pelvic, and cutaneous regions [2,3,5,7,9].

Careful microbiological examination and histopathology are the cornerstones of diagnosis. Furthermore, these microorganisms exhibit slow growth and have fastidious nutritional requirements, which poses a challenge for precise identification [10].

## Case presentation

A 56-year-old male was admitted to the emergency department for diffuse abdominal pain and thoracic pain over two weeks. This patient was working as a certified livestock technician and was regularly exposed to cattle. Relevant medical and surgical history includes diabetes mellitus; tobacco use (20 cigarettes per day since the age of 23 years); chronic alcoholic liver disease, with previous daily alcohol consumption >100 g per day (until the age of 51 years, reporting total abstinence since then); and alcoholic chronic pancreatitis, with multiple exacerbations, complicated by a pancreatic pseudocyst. Furthermore, the patient underwent a Wirsungo-gastrostomy and cholecystectomy seven years prior to admission.

Regarding the symptoms that led the patient to seek medical attention, in addition to those previously mentioned, the patient reported significant weight loss (approximately 8 kg), profuse nocturnal sweating, and anorexia, in the same period of time. Additionally, the patient developed mild dyspnea and brownish sputum, with no evidence of hemoptysis. Intermittent febrile episodes were also described, with maximal body temperature recorded at 39ºC. There were no signs of visible blood loss, gastrointestinal or urinary symptoms, peripheral edema, or skin manifestations. The patient reported no recent travel, well-water consumption, or contact with animals, other than those associated with his professional activity. 

**Figure 1:**
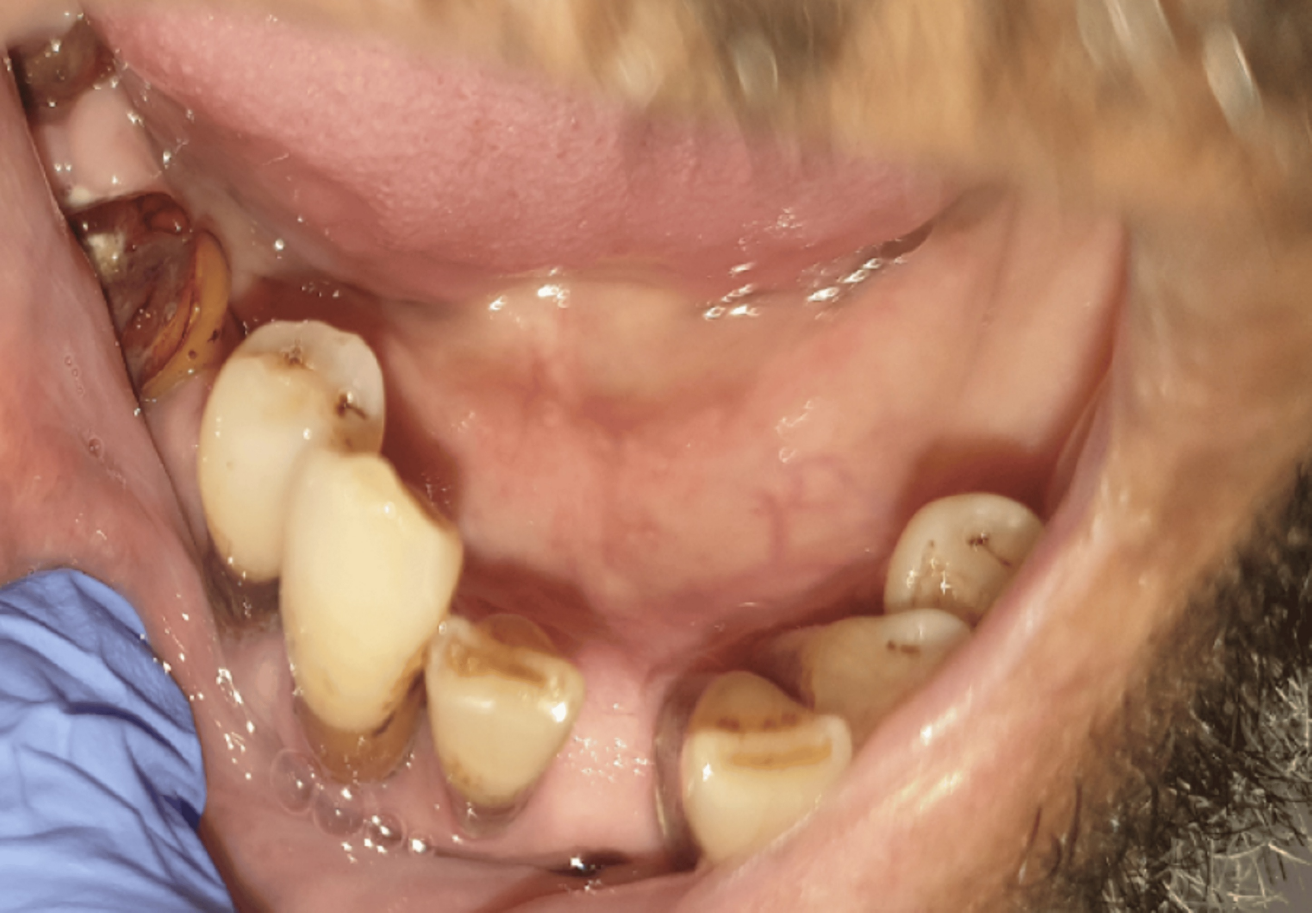
Patient’s lower dental arch with multiple caries.

Upon admission, on physical examination, the patient presented with fever (38ºC), icteric sclerae, and multiple dental caries (Figure 1). He was normotensive and without hypoxia, with an oxygen saturation of 95%. Pulmonary auscultation revealed asymmetrical breath sounds, with diminished intensity in the lower third of the right hemithorax, with no adventitious sounds. Abdominal palpation identified tenderness in the right hypochondrium, where a palpable mass extended approximately 4-5 cm below the costal margin, along the midclavicular line.

**Figure 2:**
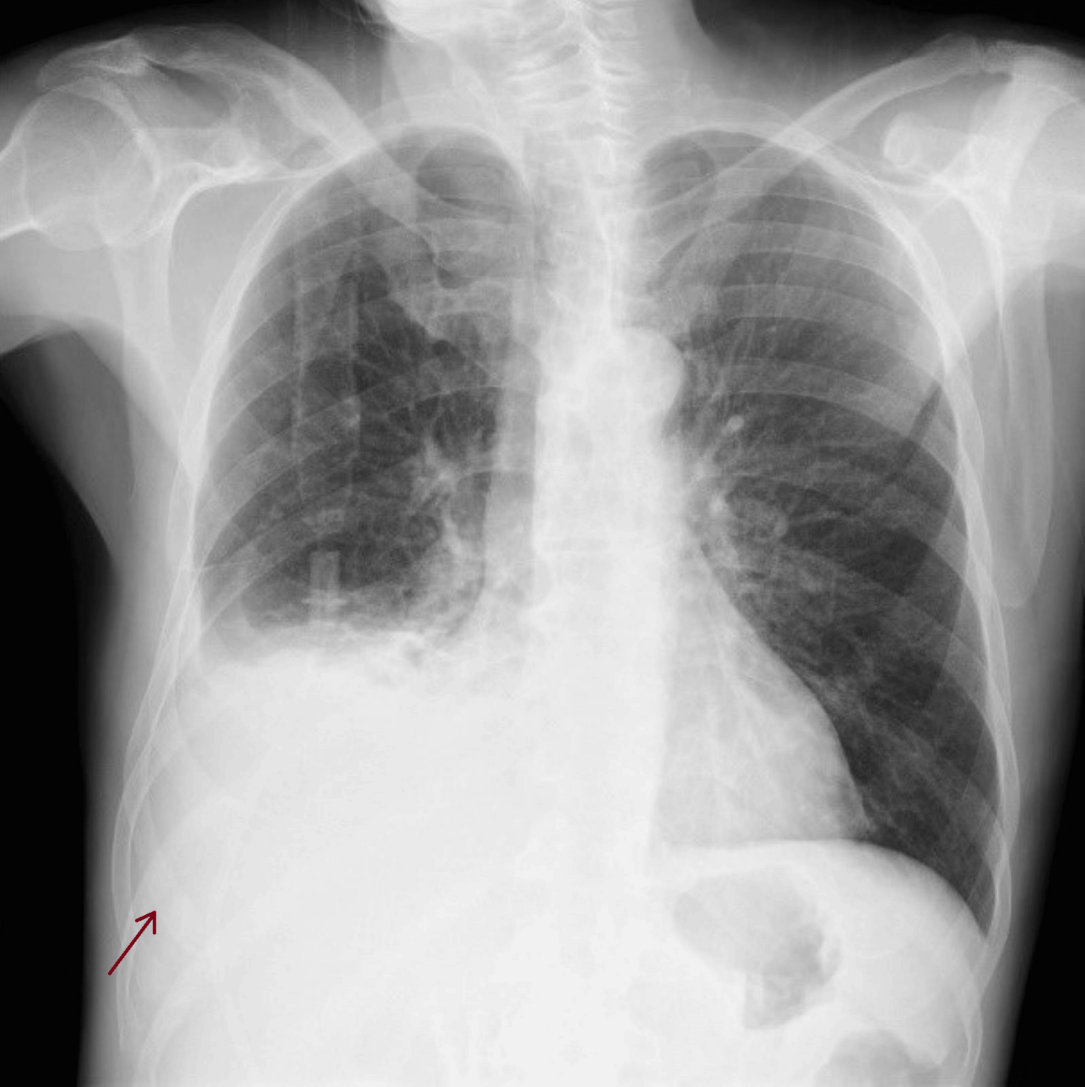
Chest X-ray performed on the day of admission suggests a right pleural effusion (arrow), posteroanterior view.

**Figure 3:**
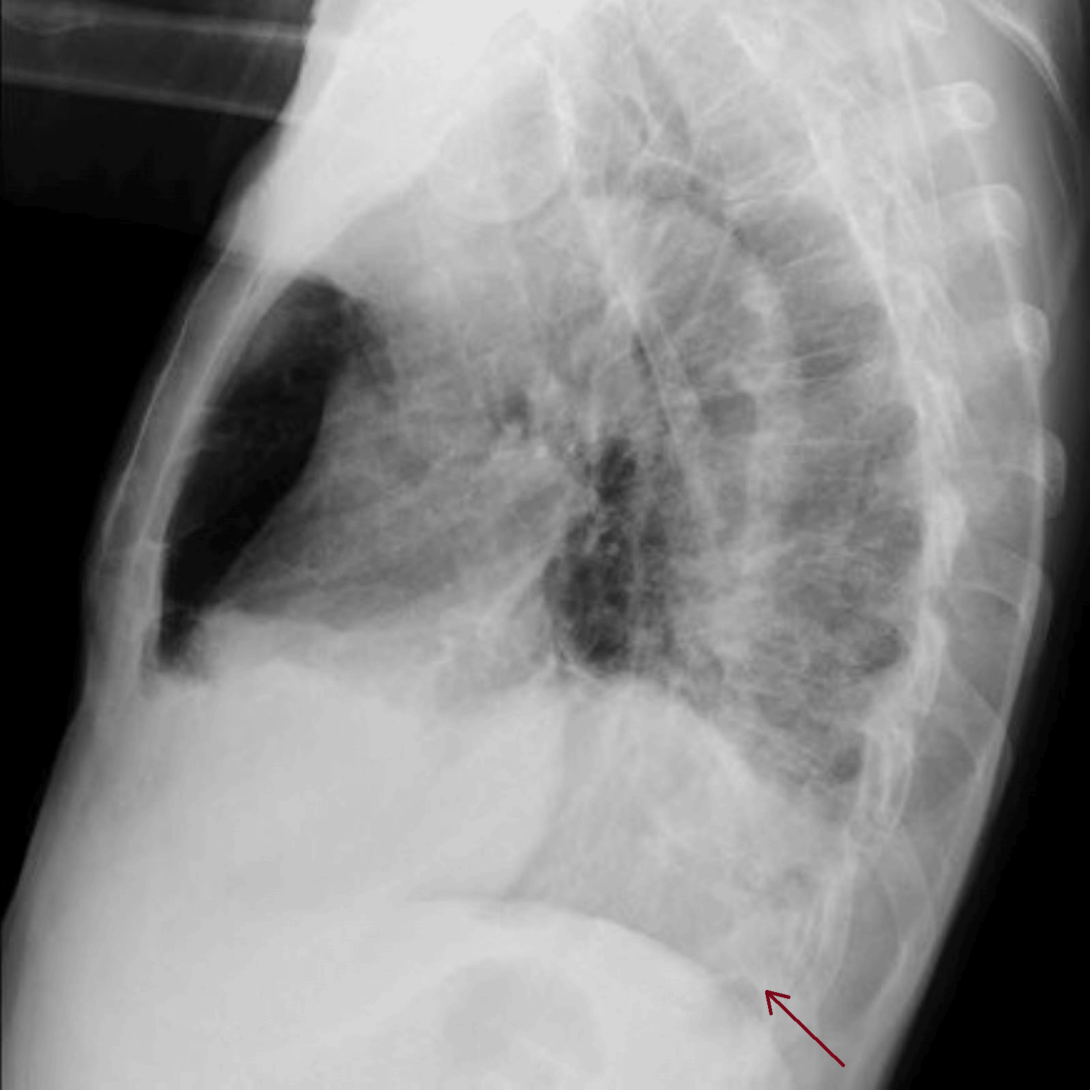
Chest X-ray performed on the day of admission suggests a right pleural effusion (arrow), lateral view.

Laboratory tests revealed normocytic anemia, leukocytosis with neutrophilia, C-reactive protein (CRP) of 38.15 mg/dL, elevated alkaline phosphatase (ALP), and gamma-glutamyl transferase (GGT) (Table 1). On the chest X-ray, hypotransparency is observed in the lower third of the right hemithorax, suggestive of a pleural effusion (Figures 2, 3).

**Table 1:** Detailed laboratory findings at the emergency department. MCV: mean corpuscular value; CHCM: cellular hemoglobin concentration mean; ALP: alkaline phosphatase; GGT: gamma-glutamyl transferase; AST: aspartate aminotransferase; ALT: alanine aminotransferase; CRP: C-reactive protein

Laboratory test	Laboratory values	Reference values
Hemoglobin	11.6 g/dL	13.2-17.2 g/dL
MCV	83.8 fL	80-96.1 fL
CHCM	35 g/dL	31.7-35.7 g/dL
White blood cells	17.98 x 10^9^/L	4.0-10.0 x 10^9^/L
Neutrophils	87.7%/15.8 x 10^9^/L	55-75%/1.5-8.0 x 10^9^/L
Platelets	545 x 10^9^/L	150-400 x 10^9^/L
Total bilirubin	0.73 mg/dL	0.3-1.2 mg/dL
Direct bilirubin	0.25 UI/L	<0.5 UI/L
ALP	543 UI/L	30-120 UI/L
GGT	357 UI/L	<55 UI/L
AST	37 UI/L	8-35 UI/L
ALT	43 UI/L	10-45 UI/L
Amylase	12 UI/L	22-80 UI/L
Lipase	8 UI/L	3-75 UI/L
CRP	38.15 mg/dL	<0.51 mg/dL

**Figure 4:**
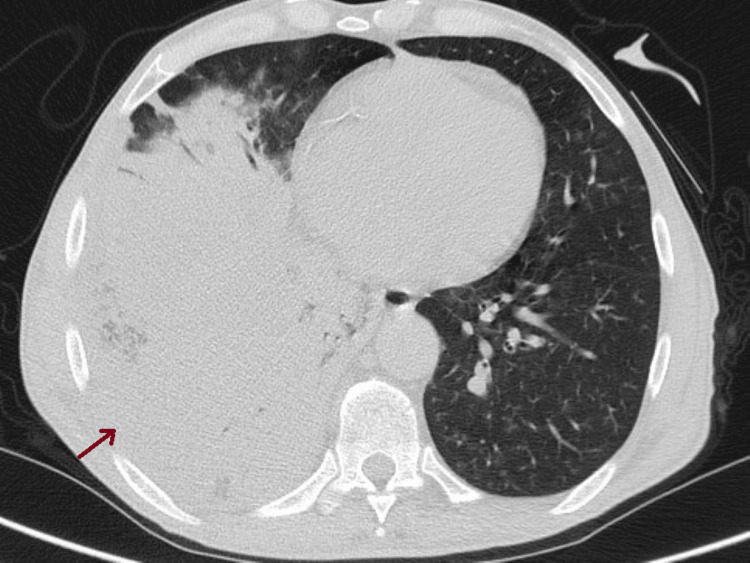
Thoracic CT scan performed upon admission shows consolidation in the right lung, with multiple nodular opacities visible (arrow).

For further clarification, a thoracic CT scan was performed, revealing the following: consolidation with an air bronchogram in the right lower and middle lobes, associated with multiple small nodular opacities and diffusely scattered ground-glass densities, predominantly in the right lung and left lower lobe, and a mild right pleural effusion (Figure 4).

**Figure 5:**
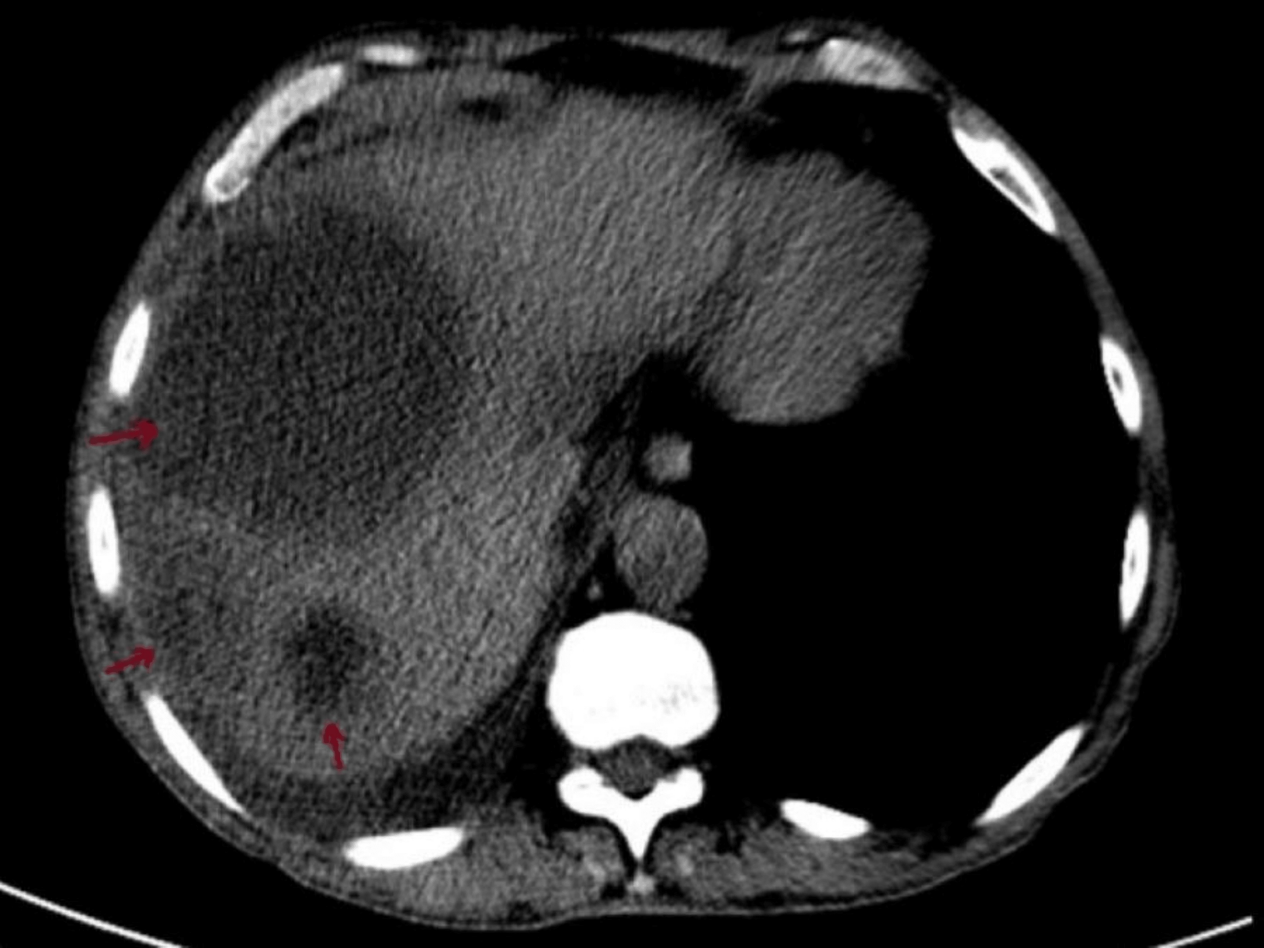
Abdominal CT scan showing nodular hepatic lesions (arrows).

**Figure 6:**
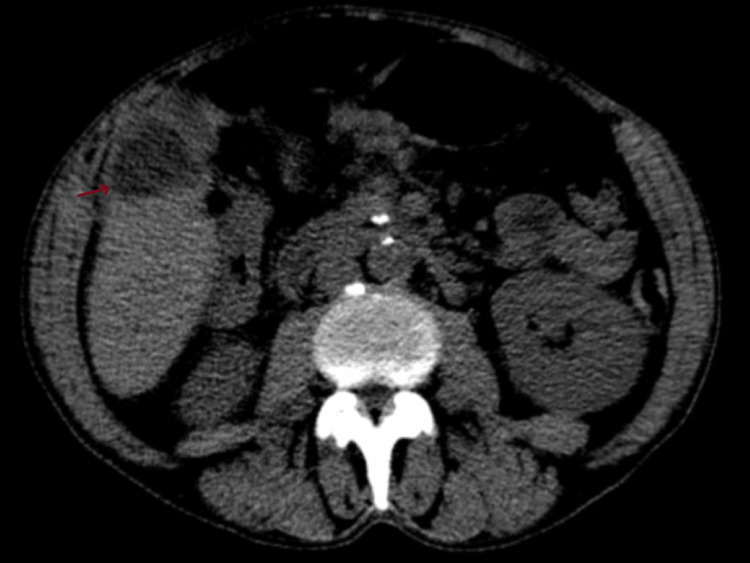
Abdominal CT scan showing nodular hepatic lesions (arrow).

Moreover, an abdominal-pelvic CT scan demonstrated multiple coarse pancreatic calcifications consistent with chronic pancreatitis; hepatomegaly with left lobe prominence; and a dominant hypodense nodular lesion in the right lobe (105x90 mm), alongside adjacent indeterminate lesions (40 mm, 39 mm, 35 mm) (Figures 5, 6).

**Figure 7:**
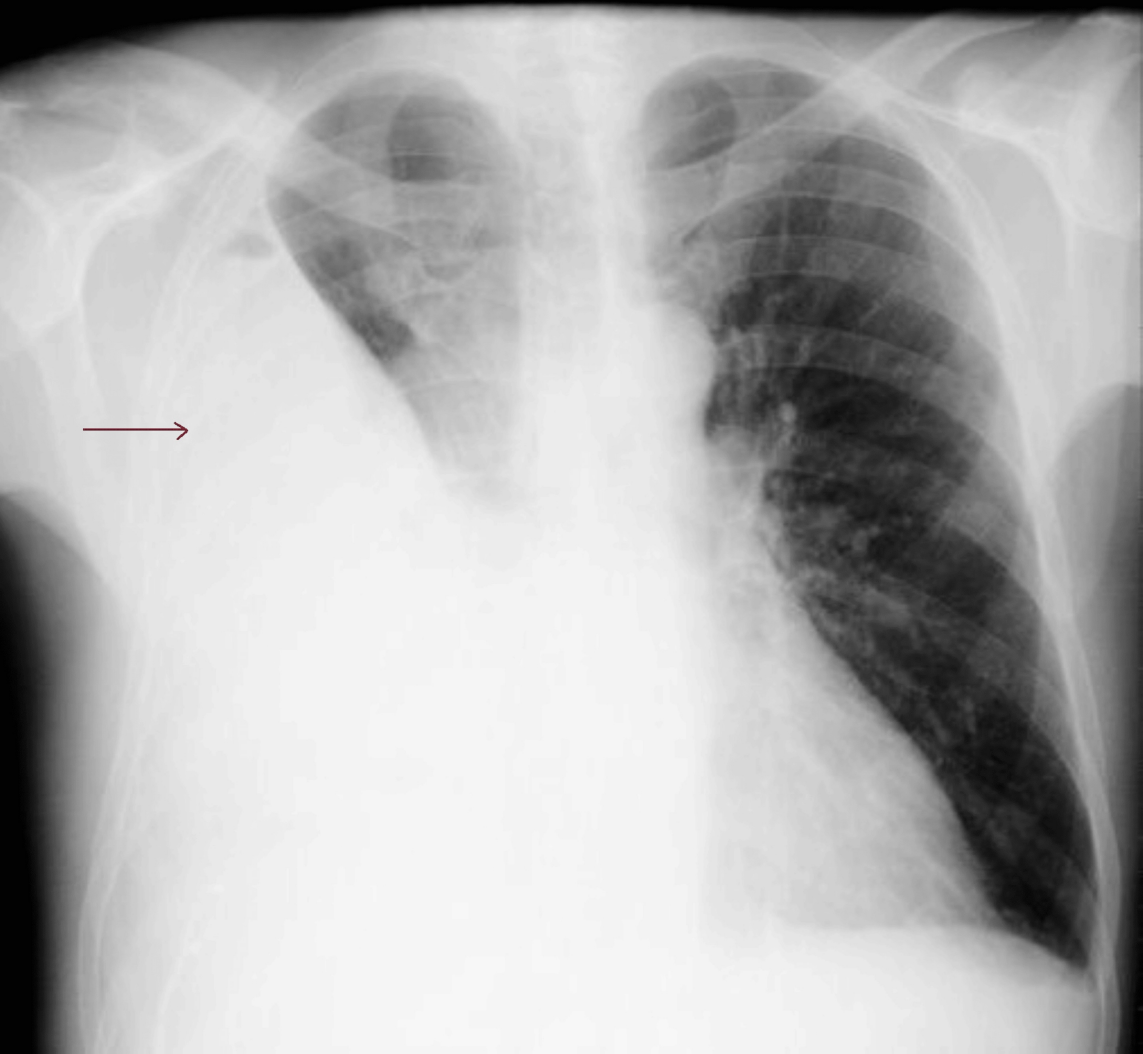
Chest X-ray performed on the third day of hospitalization shows worsening of the hypotransparency in the right hemithorax (arrow), posteroanterior view.

**Figure 8:**
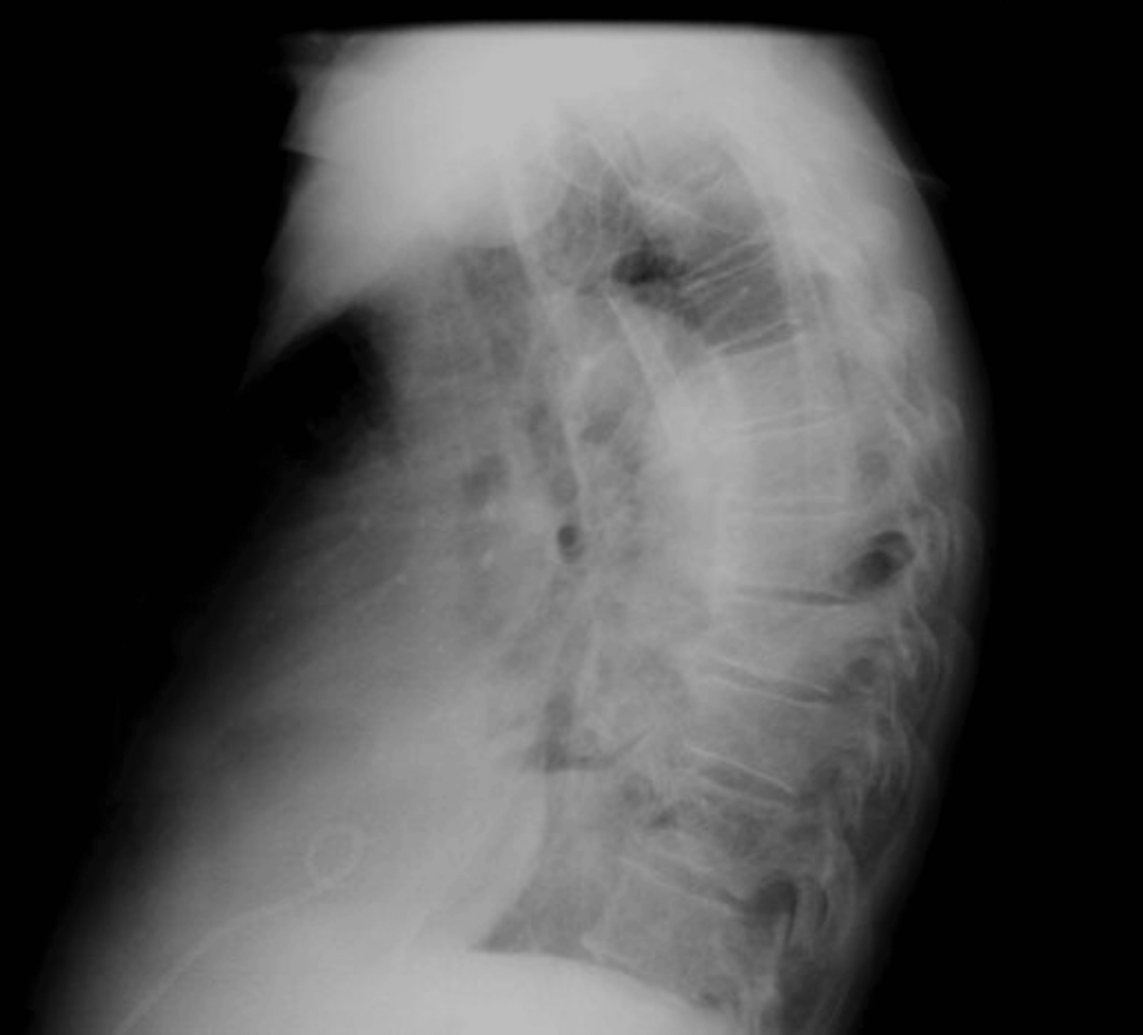
Chest X-ray performed on the third day of hospitalization shows worsening of the hypotransparency in the right hemithorax, lateral view.

Hence, based on the diagnosis of pneumonia, empirical antibiotic therapy was initiated, with ceftriaxone and azithromycin. The patient was admitted for inpatient care and started on antibiotic therapy while awaiting an MRI to further investigate the findings from the abdominal CT scan. After three days of hospitalization, the patient still presented with a fever, prompting the need for a new chest X-ray and blood tests. As seen in Figures 7, 8, there was an increase in the size of the hypotransparency in the right hemithorax.

**Figure 9:**
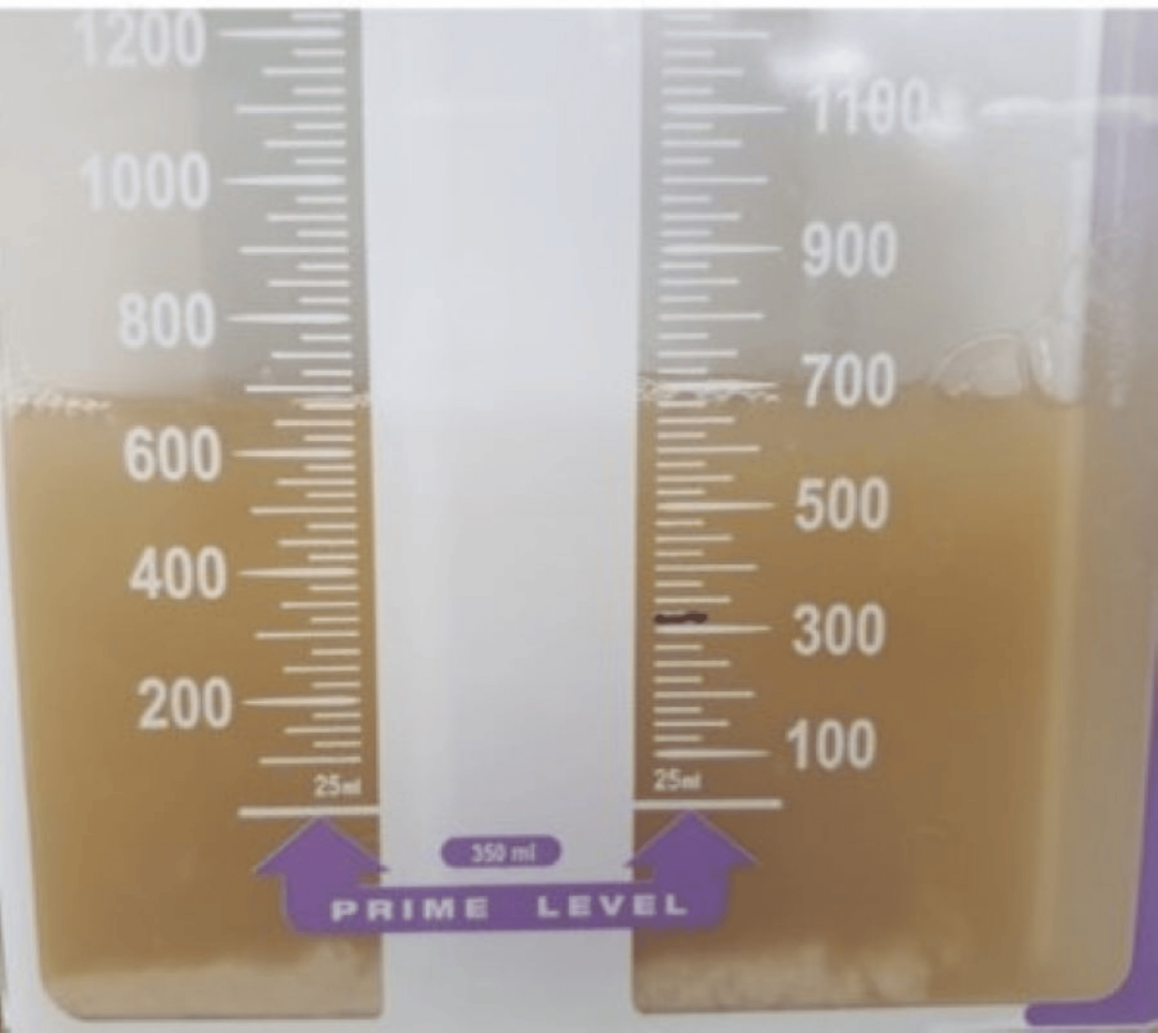
Pleural fluid drained.

Regarding laboratory findings, there was a worsening of anemia and thrombocytosis, with a decrease in CRP levels, despite a slight increase in leukocytosis (Table 2). Therefore, considering the lack of clinical and analytical response, the antibiotic therapy was switched to piperacillin/tazobactam, and thoracentesis was performed with the placement of a chest drain. In Figure 9, the purulent appearance of the drained pleural fluid is visible. Its laboratory analysis revealed characteristics consistent with empyema, as described in Table 3.

**Table 2:** Detailed laboratory findings on the third day of hospitalization. MCV: mean corpuscular value; CHCM: cellular hemoglobin concentration mean; CRP: C-reactive protein

Laboratory test	Laboratory values	Reference values
Hemoglobin	8.7 g/dL	13.2-17.2 g/dL
MCV	84.3 fL	80.1-96.1 fL
CHCM	34.2 g/dL	31.7-35.7 g/dL
White blood cells	18.61 x10^9^/L	4.0-10.0 x 10^9^/L
Neutrophils	82.4%/15.3 x10^9^/L	55-75%/1.5-8.0 x 10^9^/L
Platelets	859 x10^9^/L	150-400 x 10^9^/L
CRP	23.5 mg/dL	<0.51 mg/dL

**Table 3:** Detailed analysis of the pleural fluid. ADA: adenosine deaminase; LDH: lactate dehydrogenase

Laboratory test	Laboratory values	Reference values
pH	7.2	7.6-7.8
Total cell count	380,826 cells/mm³	1,000-3,000 cells/mm³
Leukocytes + other cells	213,726 cells/mm³	<1,000 cells/mm³
Erythrocytes	167,100 mm³	N/A
Neutrophils	87%	<25% of total leukocytes
Lymphocytes	4%	20%-50% of total leukocytes
Mononuclear cells	7%	<10% of total cells
Binucleated cells	2%	N/A
ADA	500.2 U/L	<40 U/L
Proteins	2.5 g/dL	1.0-2.0 g/dL
Albumin	38.15 g/dL	N/A
LDH	9,000 U/L	100-200 U/L
Glucose	<5 mg/dL	60-100 mg/dL

**Figure 10:**
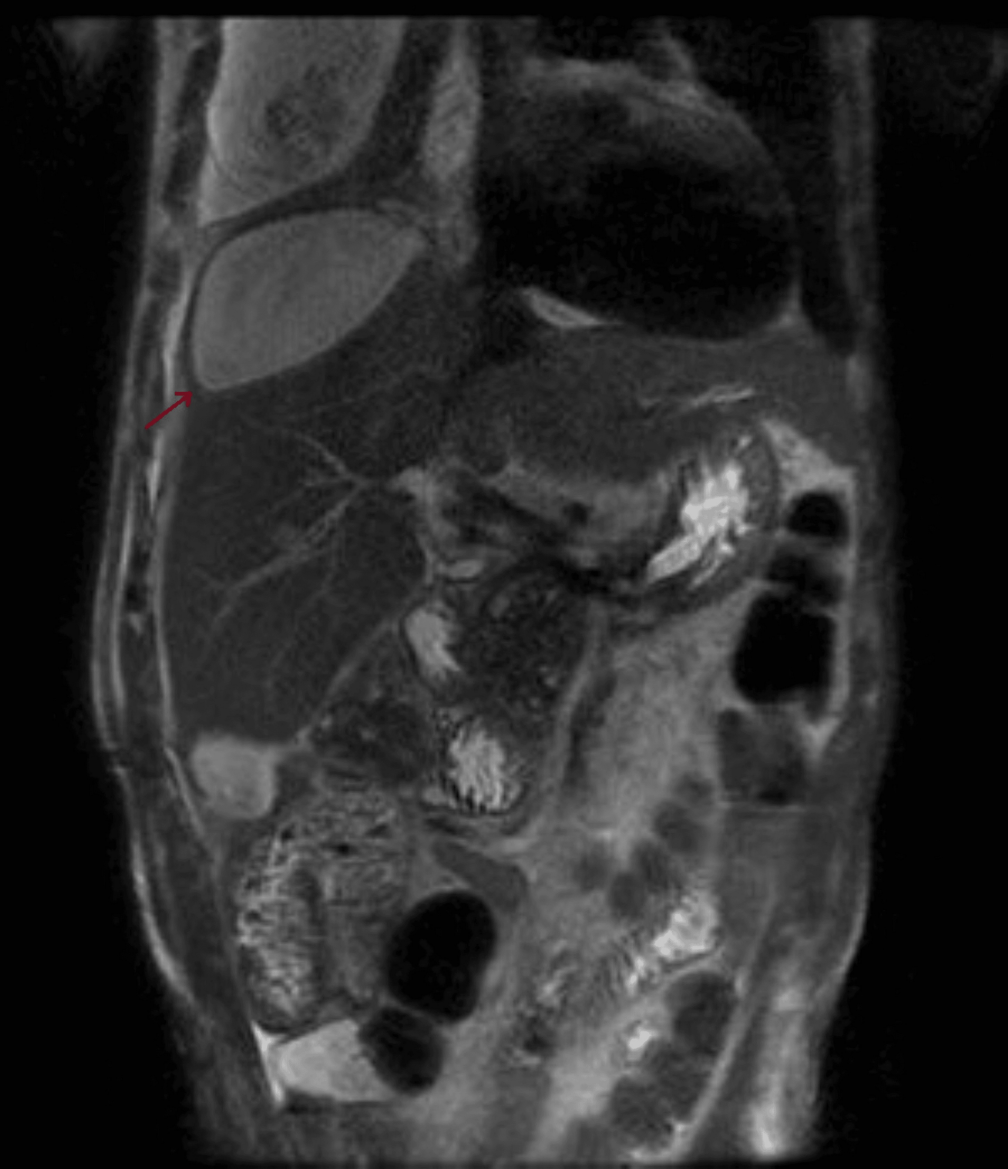
Abdominal MRI performed on the fourth day of hospitalization demonstrating numerous hepatic lesions (arrow), suggestive of hepatic abscesses, coronal view.

**Figure 11:**
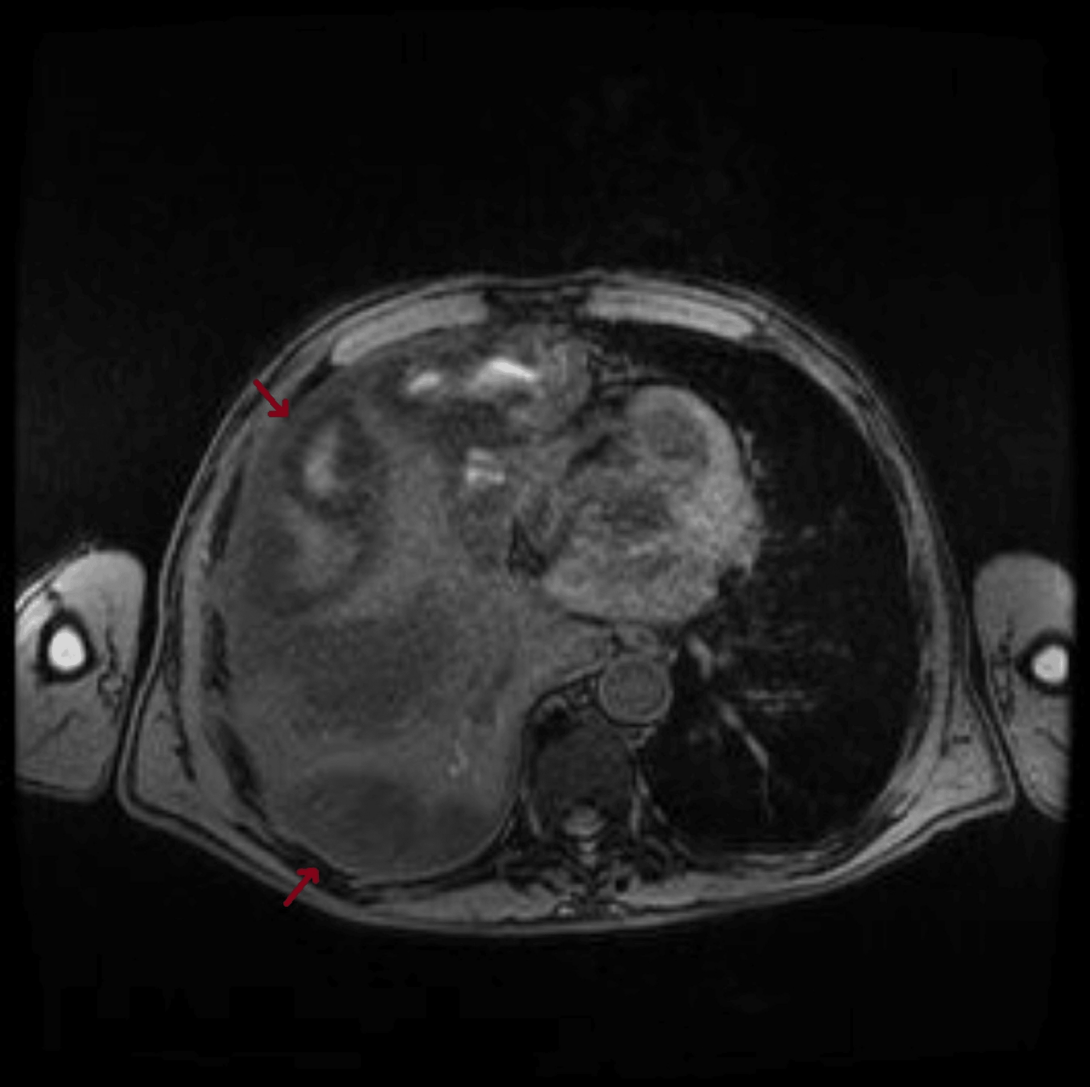
Abdominal MRI performed on the fourth day of hospitalization demonstrates numerous hepatic lesions (arrows), suggestive of hepatic abscesses, axial view.

**Figure 12:**
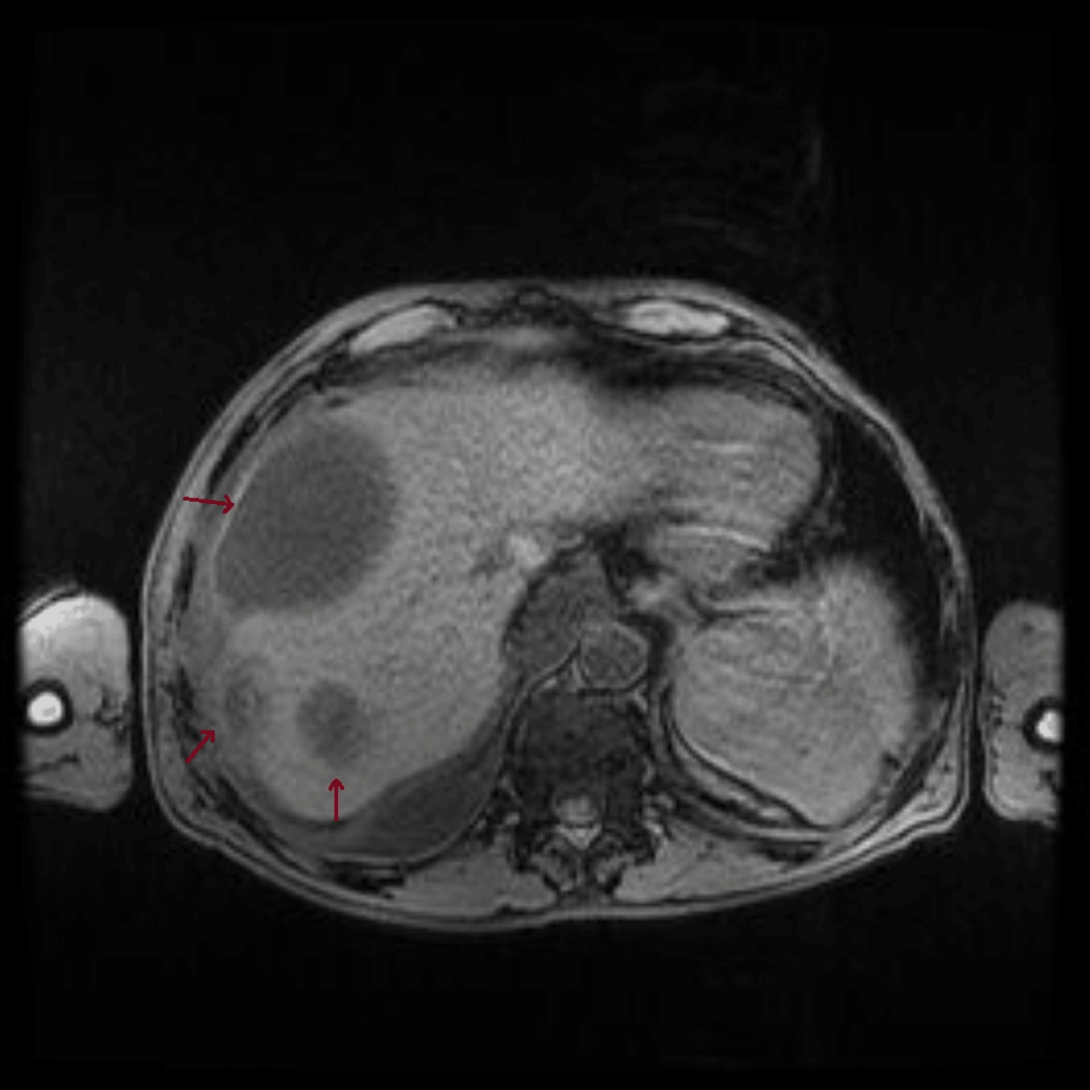
Abdominal MRI performed on the fourth day of hospitalization shows multiple hepatic lesions (arrows), suggestive of hepatic abscesses. The image shows the largest lenticular lesion centered on segment VIII, measuring approximately 10x8 cm.

Only on the fourth day of hospitalization was it possible to perform an abdominal MRI for further clarification, which revealed the following: in the right hepatic lobe, several heterogeneous subcapsular lesions were identified, the largest measuring approximately 10 x 8 cm, suggesting a predominantly liquid component, although heterogeneous. In segment VII, two other smaller lesions were delineated, measuring 28 mm and 33 mm. A peripheral lesion was also observed in the lower right quadrant, measuring 48 mm. These findings suggested multiple hepatic abscesses (Figures 10-12).

Given this information, the largest collection was drained under ultrasound guidance. During the procedure, communication was identified between the aforementioned subphrenic collection and the pleural space at the base of the right hemithorax through a fistulous opening approximately 6 mm in diameter, allowing free passage of liquid material between the two compartments. Given this new information, on the fourth day of hospitalization, the antibiotic strategy was adjusted again to include ceftriaxone and metronidazole. Meanwhile, the results of the blood cultures collected at admission became available as follows: two aerobic cultures were negative and one anaerobic culture was positive for *Actinomyces odontolyticus*.

The pleural fluid was analyzed for bacteriological examination and mycobacteria screening, both of which were negative. A bacteriological examination of the pus collected during the drainage of the hepatic abscess was also performed, with negative results. Thereby, the diagnosis of actinomycosis with pulmonary and hepatic involvement was established, and the patient continued treatment with ceftriaxone monotherapy.

A new drainage procedure was carried out on the 20th day of hospitalization. A new sample was once again collected for bacteriological analysis; however, the result was also negative. At that time, a new set of blood cultures was also collected, with no isolation of any organisms. Therefore, the only bacteriological isolation was obtained from one of the blood cultures taken upon admission. Thus, the patient completed four weeks of intravenous antibiotic therapy with ceftriaxone, showing marked improvement. Consequently, at the end of this period, the treatment was switched to oral amoxicillin.

**Figure 13:**
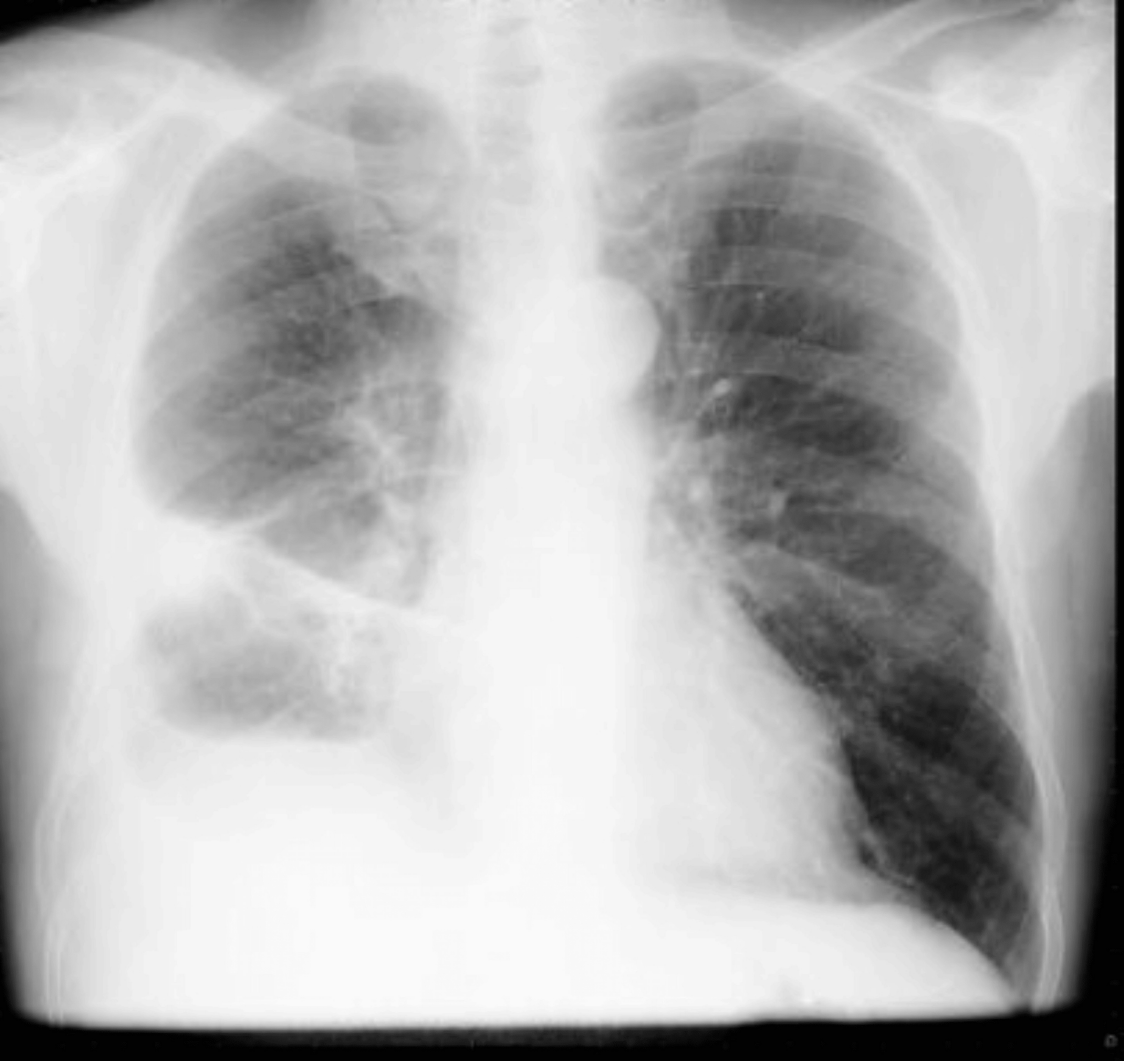
Chest X-ray on the 43rd day of hospitalization shows improvement after antibiotic therapy.

After 43 days of hospitalization, the patient showed significant clinical, analytical (Table 4), and imaging improvement (Figure 13). An abdominal ultrasound was also performed, which described only changes associated with chronic liver disease phenomena, without any lesions, thus confirming the resolution of the hepatic abscesses.

**Table 4:** Detailed laboratory findings at the 43rd day of hospitalization. MCV: mean corpuscular value; CHCM: cellular hemoglobin concentration mean; ALP: alkaline phosphatase; GGT: gamma-glutamyl transferase; AST: aspartate aminotransferase; ALT: alanine aminotransferase; CRP: C-reactive protein

Laboratory test	Laboratory values	Reference values
Hemoglobin	12.6 g/dL	13.2-17.2 g/dL
MCV	84.3 fL	80-96.1 fL
CHCM	34.2 g/dL	31.7-35.7 g/dL
White blood cells	6.85 x10^9^/L	4.0-10.0 x 10^9^/L
Neutrophils	62%/4.25 x10^9^/L	55-75%/1.5-8.0 x 10^9^/L
Platelets	346 x10^9^/L	150-400 x 10^9^/L
ALP	160 UI/L	30-120 UI/L
GGT	178 UI/L	<55 UI/L
AST	50	8-35 UI/L
ALT	42	10-45 UI/L
CRP	0.35 mg/dL	<0.51 mg/dL

Thus, the patient was discharged with instructions to continue outpatient antibiotic therapy with amoxicillin, completing a total of three months of treatment. Five months after treatment, new laboratory tests were performed, showing normalization of hepatic function parameters, with inflammatory markers remaining negative (Table 5).

**Table 5:** Detailed laboratory findings five months after treatment. MCV: mean corpuscular value; CHCM: cellular hemoglobin concentration mean; ALP: alkaline phosphatase; GGT: gamma-glutamyl transferase; AST: aspartate aminotransferase; ALT: alanine aminotransferase; CRP: C-reactive protein

Laboratory test	Laboratory values	Reference values
Hemoglobin	13.6 g/dL	13.2-17.2 g/dL
MCV	87.5 fL	80-96.1 fL
CHCM	34.8 g/dL	31.7-35.7 g/dL
White blood cells	5.38 x10^9^/L	4.0-10.0 x 10^9^/L
Neutrophils	61.9%/3.31 x10^9^/L	55-75%/1.5-8.0 x 10^9^/L
Platelets	251 x10^9^/L	150-400 x 10^9^/L
ALP	86 UI/L	30-120 UI/L
GGT	55 UI/L	<55 UI/L
AST	23 UI/L	8-35 UI/L
ALT	26 UI/L	10-45 UI/L
CRP	0.23 mg/dL	<0.51 mg/dL

**Figure 14:**
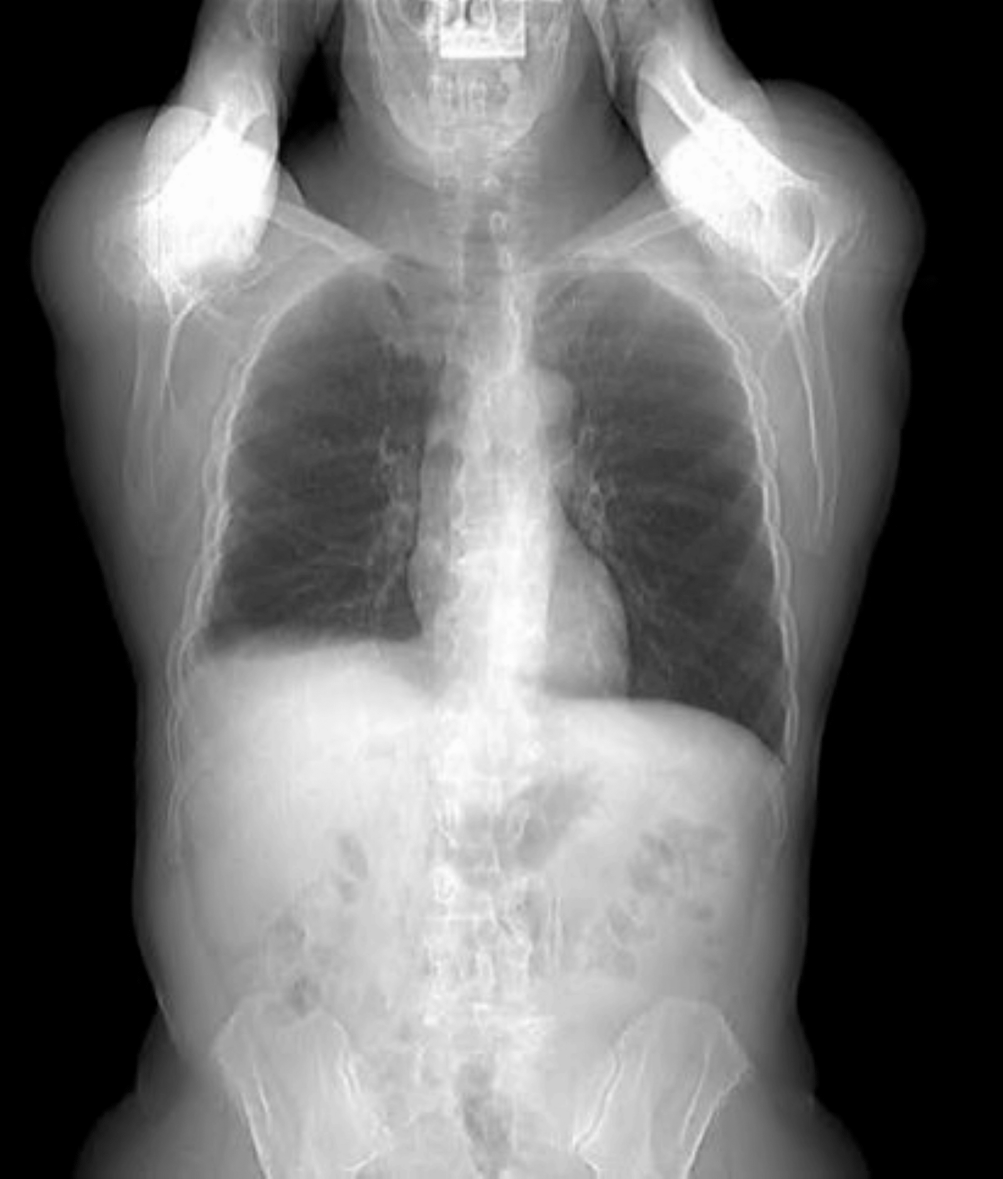
Thoracoabdominal CT scan performed five months after diagnosis (coronal view), with marked improvement after antibiotic therapy.

**Figure 15:**
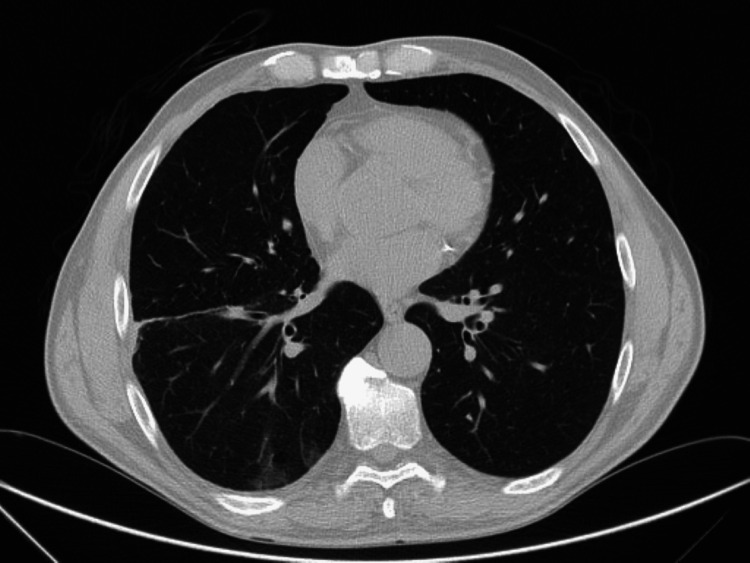
Thoracoabdominal CT scan performed five months after diagnosis, with marked improvement after antibiotic therapy (axial view).

**Figure 16:**
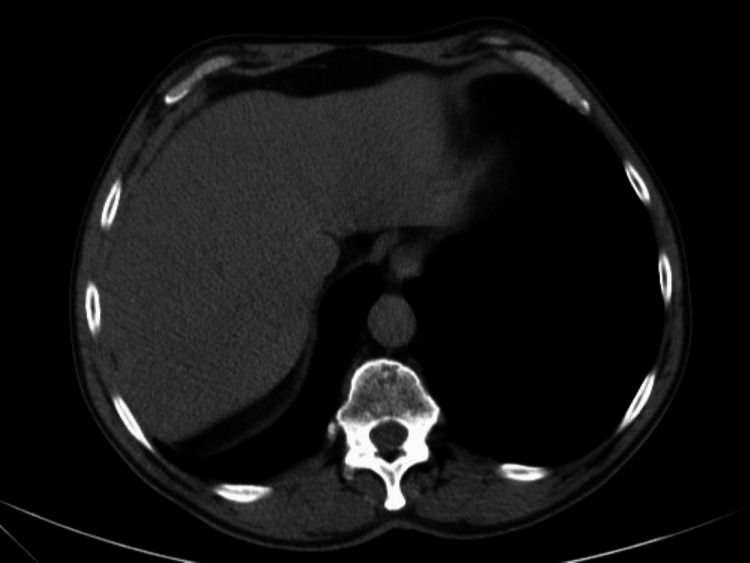
Thoracoabdominal CT scan performed five months after diagnosis, with complete resolution of the hepatic abscesses.

The patient also underwent imaging reassessment with a thoracoabdominal CT scan, which demonstrated some pulmonary fibrotic streaks and fissural thickening on the right, of a scar-like nature, along with resolution of the other abnormalities previously observed (Figures 14-16).

## Discussion

Actinomycosis poses a significant diagnostic challenge due to its rarity and the lack of specificity in its clinical and imaging presentation. In this clinical case, empirical therapy was initially initiated based only on the hypothesis of community-acquired pneumonia; however, the treatment yielded unfavorable results. The patient was also hospitalized for further investigation of the hepatic lesions, which were later identified as multiple hepatic abscesses with a fistulous connection to the pleural space.

The diagnosis was established through blood culture isolation of Actinomyces odontolyticus, highlighting the importance of microbiological investigation. Hence, this clinical scenario represents a severe and uncommon manifestation of actinomycosis, as this pathogen is most frequently associated with cervicofacial infection but, more rarely, is also capable of causing pulmonary and hepatic involvement. This underscores the versatility of Actinomyces spp., which are part of the normal oral flora but can cause serious infections, particularly when they gain access to deeper tissues through trauma or other predispositions, such as poor oral hygiene or immunocompromised states [3,7]. In this patient, with multiple dental caries, the extensive history of chronic alcohol consumption and diabetes likely predisposed him to opportunistic infections, including actinomycosis.

On the one hand, effective management involved drainage of both the hepatic abscesses and the pleural fluid collection and, on the other hand, prolonged antibiotic therapy played a major role, consistent with current recommendations. A combination of penicillin or cephalosporins with metronidazole can be administered to cover both the anaerobic nature of the bacteria and the possibility of polymicrobial infection [5,6]. This therapeutic approach was successful, with the patient demonstrating significant clinical, analytical, and imaging improvement over the course of treatment.

This case illustrates the importance of considering actinomycosis as a differential diagnosis in patients with complex infections involving multiple organ systems, particularly when common pathogens are ruled out. It also highlights the need for multidisciplinary management, combining surgical expertise and prolonged antibiotic therapy to achieve favorable outcomes [8]. Despite the challenges in diagnosing actinomycosis due to its nonspecific presentation, early recognition and targeted therapy can lead to significant clinical improvement, as seen in this patient. Additionally, the high adenosine deaminase (ADA) level in the pleural fluid, while suggestive of tuberculosis, underscores the importance of differentiating actinomycosis from other granulomatous infections.

Actinomycosis is a rare but serious infection that can present with a variety of clinical manifestations, making its diagnosis challenging. Early microbiological identification, combined with appropriate therapeutic interventions - including drainage and prolonged antibiotic therapy - is critical to achieving a positive clinical outcome. This case emphasizes the need for healthcare providers to maintain a high index of suspicion for actinomycosis, particularly in patients with unusual presentations involving multiple organs, and underscores the importance of timely intervention in managing such complex cases.

## Conclusions

This study well illustrates the importance of considering actinomycosis as a differential diagnosis of complex infectious syndromes, that might involve multiple organ systems. A high grade of suspicion, particularly in the presence of predisposing conditions, is extremely necessary. Early microbiological identification and appropriate therapeutic interventions, including drainage and prolonged antibiotic therapy, are critical for successful outcomes. Recognition in due time with all appropriate interventions significantly improves the chances of a more favorable outcome, while minimizing complications in patient care.

## References

[1] Finegold SM (2000). Anaerobic infections: general concepts. Principles and Practice of Infectious Diseases. 2000. Fifth Edition.

[2] Könönen E, Wade WG (2015). Actinomyces and related organisms in human infections. Clin Microbiol Rev.

[3] Boyanova L, Kolarov R, Mateva L, Markovska R, Mitov I (2015). Actinomycosis: a frequently forgotten disease. Future Microbiol.

[4] Hall V (2008). Actinomyces - gathering evidence of human colonization and infection. Anaerobe.

[5] Pulverer G, Schütt-Gerowitt H, Schaal KP (2003). Human cervicofacial actinomycoses: microbiological data for 1997 cases. Clin Infect Dis.

[6] Thukral R, Shrivastav K, Mathur V, Barodiya A, Shrivastav S (2017). Actinomyces: a deceptive infection of oral cavity. J Korean Assoc Oral Maxillofac Surg.

[7] Valour F, Sénéchal A, Dupieux C (2014). Actinomycosis: etiology, clinical features, diagnosis, treatment, and management. Infect Drug Resist.

[8] Gajdács M, Urbán E, Terhes G (2019). Microbiological and clinical aspects of cervicofacial Actinomyces infections: an overview. Dent J (Basel).

[9] Oostman O, Smego RA (2005). Cervicofacial Actinomycosis: diagnosis and management. Curr Infect Dis Rep.

[10] (2016). Clinical Microbiology Procedures Handbook. Fourth Edition.

